# Different Functions of Phylogenetically Distinct Bacterial Complex I Isozymes

**DOI:** 10.1128/JB.01025-15

**Published:** 2016-03-31

**Authors:** Melanie A. Spero, Joshua R. Brickner, Jordan T. Mollet, Tippapha Pisithkul, Daniel Amador-Noguez, Timothy J. Donohue

**Affiliations:** a Department of Bacteriology, University of Wisconsin—Madison, Madison, Wisconsin, USA; b Microbiology Doctoral Training Program, University of Wisconsin—Madison, Madison, Wisconsin, USA; c Great Lakes Bioenergy Research Center, University of Wisconsin—Madison, Madison, Wisconsin, USA; d Graduate Program in Cellular and Molecular Biology, University of Wisconsin—Madison, Madison, Wisconsin, USA

## Abstract

NADH:quinone oxidoreductase (complex I) is a bioenergetic enzyme that transfers electrons from NADH to quinone, conserving the energy of this reaction by contributing to the proton motive force. While the importance of NADH oxidation to mitochondrial aerobic respiration is well documented, the contribution of complex I to bacterial electron transport chains has been tested in only a few species. Here, we analyze the function of two phylogenetically distinct complex I isozymes in Rhodobacter sphaeroides, an alphaproteobacterium that contains well-characterized electron transport chains. We found that R. sphaeroides complex I activity is important for aerobic respiration and required for anaerobic dimethyl sulfoxide (DMSO) respiration (in the absence of light), photoautotrophic growth, and photoheterotrophic growth (in the absence of an external electron acceptor). Our data also provide insight into the functions of the phylogenetically distinct R. sphaeroides complex I enzymes (complex I_A_ and complex I_E_) in maintaining a cellular redox state during photoheterotrophic growth. We propose that the function of each isozyme during photoheterotrophic growth is either NADH synthesis (complex I_A_) or NADH oxidation (complex I_E_). The canonical alphaproteobacterial complex I isozyme (complex I_A_) was also shown to be important for routing electrons to nitrogenase-mediated H_2_ production, while the horizontally acquired enzyme (complex I_E_) was dispensable in this process. Unlike the singular role of complex I in mitochondria, we predict that the phylogenetically distinct complex I enzymes found across bacterial species have evolved to enhance the functions of their respective electron transport chains.

**IMPORTANCE** Cells use a proton motive force (PMF), NADH, and ATP to support numerous processes. In mitochondria, complex I uses NADH oxidation to generate a PMF, which can drive ATP synthesis. This study analyzed the function of complex I in bacteria, which contain more-diverse and more-flexible electron transport chains than mitochondria. We tested complex I function in Rhodobacter sphaeroides, a bacterium predicted to encode two phylogenetically distinct complex I isozymes. R. sphaeroides cells lacking both isozymes had growth defects during all tested modes of growth, illustrating the important function of this enzyme under diverse conditions. We conclude that the two isozymes are not functionally redundant and predict that phylogenetically distinct complex I enzymes have evolved to support the diverse lifestyles of bacteria.

## INTRODUCTION

NADH:quinone oxidoreductase (complex I) is an integral membrane electron transport chain enzyme that links catabolism to energy conservation (1). In mitochondria, complex I catalyzes NADH oxidation and the transfer of two electrons to quinone, coupling the energy of this reaction to the formation of a proton motive force (PMF) (2). NADH oxidation by mitochondrial complex I provides ∼40% of the PMF used for ATP synthesis (3). However, complex I is also widely distributed across bacteria, with genes encoding complex I subunits present in ∼50% of the sequenced species (4). Despite its occurrence in and potential contribution to prokaryotes, much less is known about the function of this enzyme in bacteria. In this study, we assess the role of complex I in Rhodobacter sphaeroides, a facultative alphaproteobacterium with well-studied electron transport chains (5, 6).

Unlike what occurs in mitochondria, bacterial electron transport chains are diverse and flexible (7). Some bacterial aerobic respiratory chains contain homologues of the mitochondrial bioenergetic enzymes (complex I, cytochrome *bc*_1_, and cytochrome *aa*_3_) (8). In contrast, other bacteria contain one or more quinol oxidases or lack some of the bioenergetic enzymes that are found in mitochondria (4, 8–10). In addition, many bacteria alter their electron transport chains in response to environmental changes. This flexibility enables bacteria to couple light energy or the oxidation of diverse electron donors (e.g., H_2_ and H_2_S) to the reduction of NAD^+^ (for the assimilation of CO_2_ or N_2_) or various electron acceptors (e.g., DMSO, NO_3^−^_, and Fe^3+^) (7, 11). Thus, complex I may function in a broader range of biological contexts than can be understood from studying its role in mitochondria.

Many pioneering studies of bacterial complex I have focused on enzyme structure and mechanism (3, 12) as opposed to its contribution to cell physiology, because the bacterial enzyme represents the “core” complex I enzyme (subunits NuoA-NuoN) (2). To date, the function of complex I has been studied in only a few bacteria. Recent phylogenetic analysis predicts the existence of 5 classes of complex I enzymes (clades A to E) across the bacterial phylogeny (4). In Escherichia coli, which encodes a clade E complex I, enzyme function is reported to be needed for growth only via anaerobic respiration using fumarate or dimethyl sulfoxide (DMSO) as a terminal electron acceptor (13). During aerobic growth, E. coli utilizes the nonbioenergetic NADH dehydrogenase NDH-2 (13). In the purple nonsulfur bacterium Rhodobacter capsulatus, which encodes a clade A isozyme, complex I is required for anaerobic growth, where the enzyme is proposed to synthesize NADH for CO_2_ or N_2_ fixation (14, 15).

This study analyzes the role of complex I in the alphaproteobacterium R. sphaeroides. This bacterium contains well-studied aerobic respiratory, anaerobic respiratory, and photosynthetic electron transport chains as well as characterized assimilatory pathways (N_2_ and CO_2_ fixation) whose function depends on reducing power that is ultimately derived from NADH (via the Rnf complex and other enzymes) (5, 6, 14, 16–19). R. sphaeroides is also one of a few bacteria predicted to encode two complex I operons (4). One of the predicted R. sphaeroides complex I isozymes (complex I_A_) is a member of clade A and is closely related to complex I enzymes found in many other alphaproteobacteria (4). The second predicted R. sphaeroides complex I isozyme (complex I_E_) is a member of clade E and is closely related to complex I enzymes found in many gammaproteobacteria, such as E. coli (4). R. sphaeroides also lacks other known NADH dehydrogenase enzymes, such as the nonbioenergetic NDH-2, or the sodium-pumping Nqr enzyme (20). Thus, R. sphaeroides provides an opportunity to assess the role(s) of phylogenetically different complex I isozymes within a single organism. We find that complex I is important during all tested modes of R. sphaeroides growth, demonstrate that the complex I_A_ and complex I_E_ enzymes are not functionally redundant, and identify metabolic conditions or cellular processes that depend partially or wholly on either or both of the complex I isozymes. Based on our findings, we present a model in which these and possibly other phylogenetically distinct complex I isozymes have evolved to function in diverse bacterial electron transport chains.

## MATERIALS AND METHODS

### Bacterial growth.

Wild-type R. sphaeroides strain 2.4.1 and mutant strains were grown at 30°C in Sistrom's minimal medium (SMM), using succinate and ammonium as the carbon and nitrogen sources, respectively (21), unless other carbon (fumarate, pyruvate, malate, or dl-lactate) or nitrogen (glutamate) sources were added at concentrations previously described (16). Aerobic cultures were shaken in flasks or 96-well plates, using the optical density at 595 nm (OD_595_) to monitor cell density. Photoheterotrophic cultures were grown in filled 17-ml screw-cap tubes (10-W/m^2^ light intensity), containing 100 mM DMSO when indicated, and used a Klett-Summerson colorimeter (number 66 filter) to measure cell density. To test photoautotrophic growth, SMM plates lacking succinate, aspartate, and glutamate were illuminated (10 W/m^2^) in anaerobic jars under a CO_2_-H_2_ environment (BBL GasPak Plus Anaerobic System Envelopes with Palladium Catalyst; BD Diagnostic Systems). To test anaerobic respiratory growth, SMM plates containing 100 mM DMSO were placed in dark anaerobic jars. E. coli strains were grown at 37°C in Luria broth, using E. coli strain DH5α as a plasmid host and strain S17-1 as a conjugal host for matings with R. sphaeroides.

### Mutant construction.

R. sphaeroides complex I mutants contained in-frame, markerless deletions (22). The Δcomplex I_A_ and Δcomplex I_E_ strains were generated by deleting the gene encoding the NuoG subunit from each respective operon, as NuoG is required for complex I function (23, 24). Specifically, complex I_A_ *nuoG* (RSP2521) plus ∼1 kbp flanking DNA was amplified with primers 5′-TATCGTCGACTGAAGCTCTTCGCCATGTCG-3′ (underlining indicates the SalI restriction site) and 5′-TATCGGATCCCAGCGCGGAGATGAAGAACA-3′ (underlining indicates the BamHI site), while complex I_E_ *nuoG* (RSP0105) plus ∼1 kbp flanking DNA was amplified with primers 5′ GATCGTCGACTTCAAGGACCGCTTCCTGCT-3′ (SalI site underlined) and 5′-GATCGAATTCACCTTCCAGGCAAAGGAGAT-3′ (EcoRI site underlined). The amplified products were digested with the appropriate restriction enzymes and ligated into pK18*mobsacB*. Gene deletions were created in pK18*mobsacB* using PCR primers within the ∼1-kbp flanking regions. The primers used to generate pK18*mobsacB*-ΔRSP2521 and pK18*mobsacB*-ΔRSP0105 were 5′-GTTACATATGGGCGCCATCGACCTCGAC-3′ and 5′-GTTACATATGGGGGTGCGGCCGGCGG-3′ and 5′-GTTACATATGGCCCGCTCTCCTTCGG-3′ and 5′-GTTACATATGGTGCAGGACTCTTCCT-3′ (NdeI site underlined). The amplified product was treated with NdeI and circularized by ligation. The resulting plasmids were mobilized from E. coli S17-1 into R. sphaeroides via conjugation, and cells that had integrated the plasmid into the genome were identified by plating on SMM with kanamycin under aerobic conditions. Colonies were streaked for purity, and cells were resuspended in SMM and then plated under aerobic conditions onto SMM with 10% sucrose. Isolated colonies were transferred onto SMM with kanamycin and SMM with 10% sucrose plates to screen for cells that had lost the plasmid (no growth on kanamycin, growth on sucrose). Strains that lost the plasmid and contained the desired gene deletion were identified by PCR. DNA sequence analysis of the appropriate genomic region was used to confirm that candidate strains contained the desired mutation.

### qRT-PCR assays.

Published methods were used to isolate RNA from photoheterotrophically grown cells (∼1.8 × 10^9^ cells/ml) and for cDNA synthesis (19). Triplicate reverse transcription-quantitative PCR (qRT-PCR) assays were conducted for each biological replicate (SYBR green JumpStart *Taq* ReadyMix; Sigma-Aldrich). Relative mRNA levels were calculated by the 2^−ΔΔ*CT*^ method (where *C_T_* is threshold cycle) with efficiency correction, using *rpoZ* mRNA levels for normalization (25). Sequences for the amplification primers were 5′-TGCGAGAGTTTCTTCCCATCGTCA-3′ and 5′-CGTCGAAGGCATTGAAACCGCATT-3′ (*nuoA*, complex I_A_ operon), 5′-TATTTCCTCGTGGCCGTCTTCT-3′ and 5′-GCAGCACGAGGATGAAGATGGTG-3′ (*nuoA*, complex I_E_ operon), 5′-TTGAAGACTGCGTTGACAAGGTCC-3′ and 5′-GTTCTTGTCATTGTCGCGGTCGAT-3′ (*rpoZ*), and 5′-CGGCATTCGGTCGTCTTTA-3′ and 5′-GTTCAGAGGCTGGAACGG-3′ (*bchM*).

### H_2_ production.

The gas production (AER-200 respirometer; Challenge Technology, Springdale, AR) and composition from 20 ml photoheterotrophic, H_2_-producing cultures (containing 100 mM DMSO to correct for growth defects) were measured as described previously (16, 26).

### Pyridine nucleotide measurements.

NAD^+^/NADH levels were measured in exponential-phase photoheterotrophic cells (27). In an anaerobic chamber, cells were collected by rapid filtration onto a nylon filter disc (catalog number HNWP04700; Millipore Corp.). The filters were immediately submerged into 1.5 ml of −20°C acetonitrile-methanol-water (40:40:20) to quench metabolism and extract metabolites. Outside the anaerobic chamber, metabolites and cell debris were washed from filters before the solution was centrifuged at 20,000 × *g* for 5 min at 4°C. Supernatants were dried under N_2_ and resuspended in liquid chromatography-mass spectrometry (LC-MS) grade water (Sigma-Aldrich). Samples were analyzed (28) using a high-pressure liquid chromatography tandem mass spectrometry (HPLC-MS/MS) system consisting of a Dionex ultrahigh-pressure liquid chromatography (UHPLC) system coupled by electrospray ionization (ESI; negative mode) to a hybrid quadrupole-high-resolution MS (Q Exactive orbitrap; Thermo Scientific) operated in full-scan mode for mass-based compound identification. Liquid chromatography separation was achieved using either a Synergi Fusion-RP 100A (100 by 2 mm, 2.5-μm particle size; Phenomenex, Torrance, CA) or an Acquity UPLC BEH C_18_ (2.1 by 100 mm, 1.7-μm particle size) column. Solvent A was 97:3 water-methanol with 10 mM tributylamine (TBA) and 10 mM acetic acid, pH ∼8.2; solvent B was 100% methanol. Total run time was 14.5 min. Flow rate was 200 μl/min. Solvent A was 97:3 water-methanol with 10 mM TBA and 10 mM acetic acid, pH ∼8.2; solvent B was 100% methanol. The gradient was as follows: 0 min, 5% B; 1.5 min, 5% B, 11.5 min, 95% B; 12.5 min, 95% B; 13 min, 5% B; 14.5 min, 5% B. Other LC parameters were as follows: autosampler temperature, 4°C; injection volume, 5 μl; and column temperature, 25°C. Metabolite identification used a metabolomics analysis and visualization engine (29, 30).

### Bacteriochlorophyll and biomass quantification.

To measure bacteriochlorophyll (31), cells were harvested, suspended in 100 μl of water, and added to 4.9 ml of 7:2 acetone-methanol, while keeping samples in the dark. Samples were incubated at room temperature for 10 min and centrifuged at 10,000 × *g* for 10 min, and bacteriochlorophyll was quantified by measuring the absorbance of the supernatant at 775 nm using a millimolar extinction coefficient of 75 (32). Whole-cell protein was quantified using the Bradford assay with bovine serum albumin (BSA) as a standard (Bio-Rad).

To measure cell dry weight, 150 ml phototrophic cultures supplemented with 100 mM DMSO was centrifuged, and the pellets were dried at 95°C and weighed on an analytical balance.

## RESULTS

### Complex I activity is required for anaerobic growth of R. sphaeroides.

**FIG 1:**
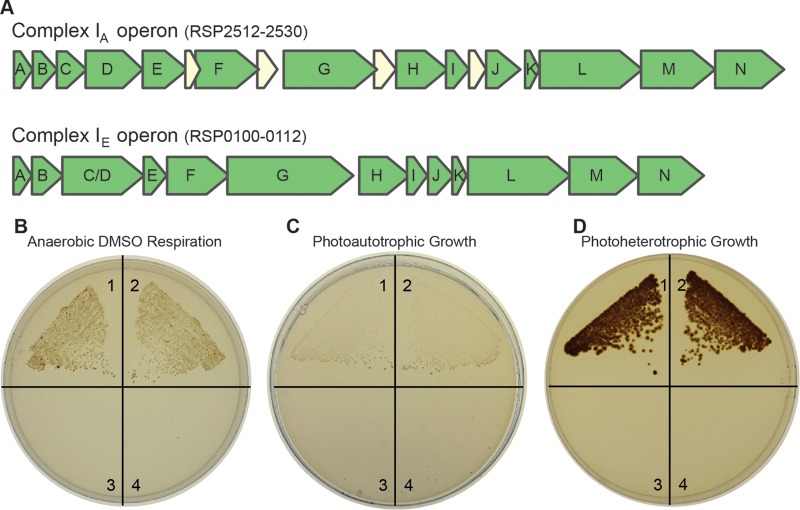
Anaerobic growth of R. sphaeroides wild-type and complex I mutant strains. (A) The two complex I-encoding operons of R. sphaeroides. (B) Cells growing by anaerobic DMSO respiration in the dark (with succinate as the carbon source); (C) cells growing photoautotrophically (anaerobic in the light, with CO_2_ and H_2_ as the carbon and electron sources); (D) cells growing photoheterotrophically (anaerobic in the light, with succinate as the carbon source). Each quadrant contains a different strain: 1, wild type; 2, Δcomplex I_E_ strain; 3, Δcomplex I_A_ strain; 4, Δcomplex I_A_/Δcomplex I_E_ strain.

The R. sphaeroides genome has two operons that encode complex I homologues (Fig. 1A). One predicted isozyme (complex I_A_) is a prototype member of complex I clade A that is typically found in alphaproteobacteria, and the second putative enzyme (complex I_E_) is a member of complex I clade E that is found in many gammaproteobacteria, such as E. coli (4). Previous studies show that the genes encoding complex I_A_ are transcribed under aerobic and anaerobic (photoheterotrophic and DMSO respiratory) growth conditions. Genes encoding complex I_E_ are transcribed only under anaerobic (photoheterotrophic and DMSO respiratory) conditions, since their transcription is dependent on either FnrL or the singlet oxygen stress response factor RpoH_II_ (33–36). While the transcript levels suggest that both R. sphaeroides operons are expressed under anaerobic conditions, no published work has examined the relative function of each complex I isozyme.

To test the role of complex I in R. sphaeroides, we analyzed strains containing an in-frame deletion in the *nuoG* gene of one or both complex I operons. We inactivated *nuoG* because this gene product chelates several iron-sulfur clusters and is involved in NADH binding/oxidation (1) and because loss of NuoG inactivates the enzyme in other organisms (23, 24). Consequently, the double mutant strain (Δcomplex I_A_/Δcomplex I_E_ strain) should lack all complex I activity, while the single mutant strains, Δcomplex I_A_ and Δcomplex I_E_ strains, synthesize only one of the two isozymes. Based on previous work in E. coli (13) or R. capsulatus (14), we expected to find growth defects in one or more of the R. sphaeroides complex I mutants under anaerobic conditions.

**FIG 2:**
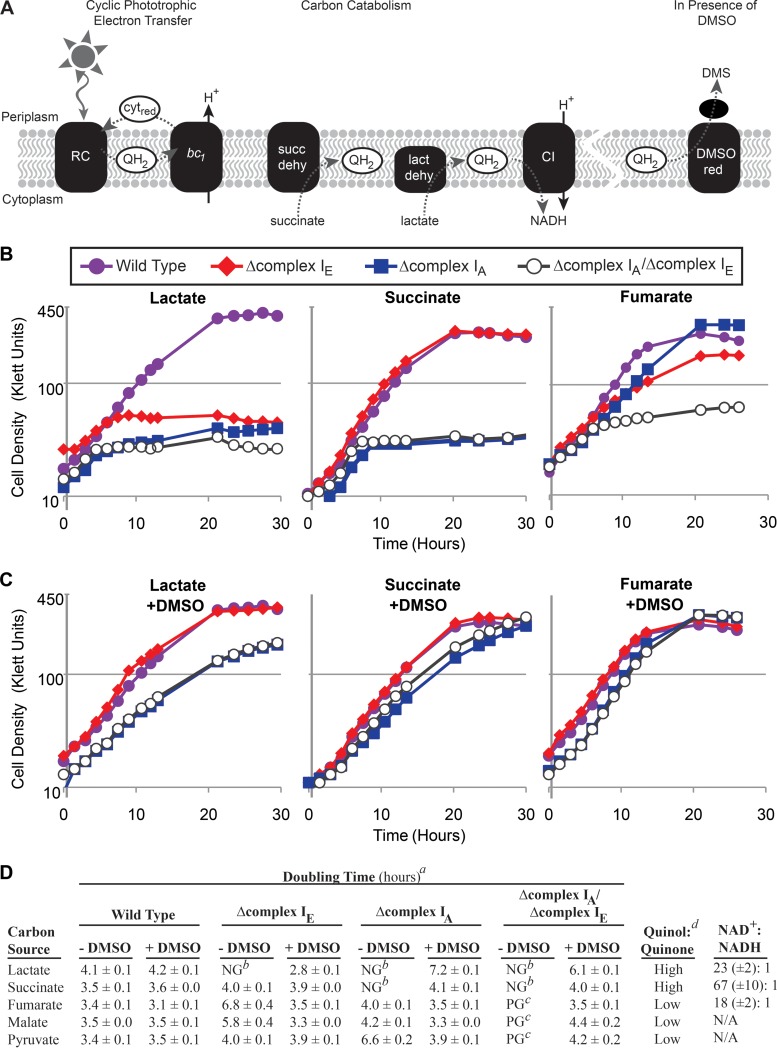
Photoheterotrophic growth of wild-type and complex I mutant strains. (A) Membrane-associated electron transfer reactions in photoheterotrophic R. sphaeroides cells. Dashed lines indicate electron transfer reactions. During cyclic phototrophic electron transfer, the light-excited reaction center (RC) produces quinol (QH_2_), which carries electrons to the cytochrome *bc_1_* complex (*bc_1_*). Electrons are transferred to the cytochrome (cyt_red_), producing a proton motive force (H^+^), and these electrons are carried back to the reaction center. Quinol is also produced via carbon catabolism by enzymes like succinate or lactate dehydrogenase (succ dehy and lact dehy, respectively). Electrons from the quinol pool can be used to synthesize NADH (via complex I [CI]) or to reduce DMSO (via DMSO reductase [DMSO red]). (B) Photoheterotrophic growth of wild-type and complex I mutant strains with lactate, succinate, or fumarate as the carbon source. Shown are representative curves for each strain from ≥3 replicates. (C) Strains were grown photoheterotrophically with the indicated carbon sources in the presence of the external electron acceptor DMSO (100 mM). Shown are representative curves for each strain from ≥3 replicates. (D) Summary of photoheterotrophic growth on different carbon sources. Superscripts: *a*, calculated doubling times (from ≥3 replicates), including standard error; *b*, NG (no growth) indicates that the average maximum cell density was <20% of the average maximum cell density of wild-type cultures under the same conditions; *c*, PG (poor growth) indicates that the average maximum cell density was 20 to 40% of the average maximum cell density of wild-type cultures under the same conditions; *d*, the relative quinol/quinone ratio of wild-type cells grown on the indicated carbon source as predicted by the R. sphaeroides metabolic model (6, 18) (see Table S2 in the supplemental material). The last column shows NAD^+^/NADH ratios measured in wild-type cells grown photoheterotrophically on the indicated carbon source (shown are the averages of ≥10 replicate measurements, including standard error).

To test this hypothesis, we compared the growths of wild-type and complex I mutant strains under dark anaerobic conditions in the presence of the electron acceptor DMSO (with succinate as the carbon source). Under these conditions, growth was observed for both wild-type and Δcomplex I_E_ strains but not for the Δcomplex I_A_ and Δcomplex I_A_/Δcomplex I_E_ (double mutant) strains (Fig. 1B). We also tested the contribution of individual complex I isozymes to photoautotrophic growth, where H_2_ serves as an electron donor (via a quinone-dependent uptake hydrogenase [37]) and CO_2_ serves as the sole carbon source. We again found that both the wild-type and the Δcomplex I_E_ strains grew photoautotrophically, while the Δcomplex I_A_ and the double mutant strains did not grow (Fig. 1C). When the same strains were grown photoheterotrophically (anaerobically in the light with succinate and ammonium as the carbon and nitrogen sources, respectively), we also found that the wild-type and Δcomplex I_E_ strains were able to grow, while Δcomplex I_A_ and the double mutant strains were not able to grow (Fig. 1D). Thus, we found that complex I_A_ is both necessary and sufficient to support growth by anaerobic DMSO respiration, photoautotrophic growth, and photoheterotrophic growth using succinate as a carbon source. By considering growth of the double complex I mutant, our results show that some complex I activity is required for growth under all tested anaerobic conditions in R. sphaeroides (except photoheterotrophic growth in the presence of an electron acceptor) (Fig. 2; see also below). Further experiments (see below) help uncover distinct roles for each complex I isozyme during photoheterotrophic growth, where the contribution of each isozyme depends on the provided carbon source. These findings lead to the prediction that each isozyme provides a specific function (NADH oxidation versus synthesis) during photoheterotrophic growth (see Results and Discussion).

### Functions of complex I isozymes during photoheterotrophic growth with different carbon sources.

The above-described experiments did not identify any condition under which the Δcomplex I_E_ mutant exhibited a growth defect (Fig. 1). One reason for such an observation might be that the complex I_E_ isozyme is nonfunctional. To address this possibility, we sought to identify one or more conditions under which complex I_E_ activity was important for growth.

We focused on photoheterotrophic growth (anaerobic conditions in the light) because the operon encoding complex I_E_ is maximally expressed under anaerobic conditions (33, 36). We found that the complex I_E_ and the complex I_A_ mutants each exhibited growth phenotypes when grown photoheterotrophically with different carbon sources (Fig. 2B). We found that the Δcomplex I_E_ strain was able to grow photoheterotrophically with succinate but unable to grow with lactate and grew more slowly than did the wild type with fumarate and malate as carbon sources (Fig. 2B and D). We observed a different set of phenotypes with the Δcomplex I_A_ mutant strain, which was unable to grow photoheterotrophically when lactate or succinate was provided as the carbon source, and the strain grew more slowly than the wild type when pyruvate was the carbon source (Fig. 2B and D). The identification of conditions under which either complex I_A_ or complex I_E_ was important for photoheterotrophic growth supports the hypothesis that each operon encodes an active complex I enzyme. Additionally, the double mutant strain was unable to grow normally during photoheterotrophic growth conditions with all tested carbon sources (Fig. 2B and D), unless the culture was supplemented with DMSO (see below).

**FIG 3:**
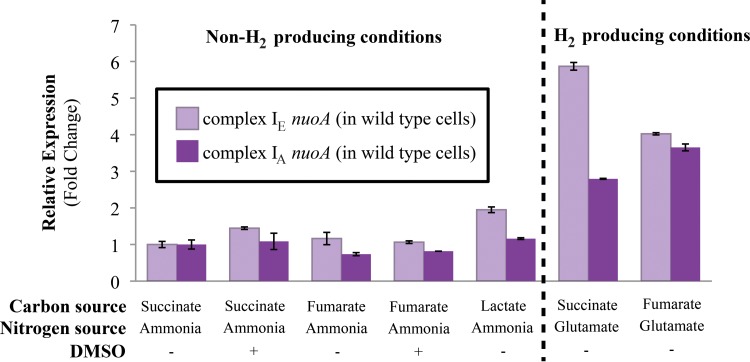
Complex I *nuoA* transcript levels in wild-type cells. Transcript levels of the *nuoA* gene from the complex I_A_ or complex I_E_ operon in wild-type cells grown photoheterotrophically with the indicated carbon and nitrogen sources, and in the presence or absence of 100 mM DMSO. Conditions under which glutamate is the provided nitrogen source are H_2_-producing conditions. Fold change values represent expressions relative to wild-type cells grown photoheterotrophically with succinate, ammonia, and in the absence of DMSO, where the relative expression of the *nuoA* transcript from the complex I_A_ or complex I_E_ operon was set to 1 (transcript levels were normalized to the “housekeeping gene,” *rpoZ*). Relative expression levels were quantified from 3 replicates and include standard errors.

To ensure that the photoheterotrophic growth defects of individual complex I mutants were not complicated by changes in the expression of the other operon, we monitored *nuoA* transcript levels by quantitative RT-PCR in both wild-type and mutant strains during photoheterotrophic growth on different carbon sources. We found that *nuoA* transcript levels showed little variation in wild-type (Fig. 3) or mutant strains grown photoheterotrophically on different carbon sources (see Table S1 in the supplemental material), suggesting that expression of individual complex I operons did not increase to compensate for the loss of the other isozyme. The lack of significant change in *nuoA* transcript levels in mutant strains supports our hypothesis that the observed photoheterotrophic growth phenotypes reflect the contribution of each isozyme. Below we describe experiments performed to explain the photoheterotrophic growth phenotypes of mutants lacking the complex I_A_ or complex I_E_ enzymes.

### The need for complex I activity is linked to the oxidation-reduction state of electron carriers.

In R. capsulatus, it is proposed that the single complex I_A_-like enzyme of this bacterium uses the PMF to synthesize NADH under photoheterotrophic conditions, which is important for preventing overreduction of the quinone pool (14, 38) (Fig. 2A). If this were also true in R. sphaeroides, the inability of complex I mutants to grow photoheterotrophically could be due to the formation of overreduced electron carrier pools in the absence of this enzyme. To test this hypothesis, we took advantage of the ability of DMSO reductase to accept electrons from the quinone pool in purple nonsulfur bacteria (5, 39). If the above hypothesis is correct, the addition of DMSO to photoheterotrophic cultures may provide a way to oxidize quinone and possibly restore growth to the complex I mutants (Fig. 2A). Indeed, the photoheterotrophic growth defects of the complex I mutant strains were partially or fully rescued by the addition of DMSO (Fig. 2C). It is important to note that during photoheterotrophic growth (anaerobic conditions and light), DMSO is known to serve as an “electron sink,” an important electron-accepting pathway that allows cells to balance overreduced electron carrier pools (e.g., NAD^+^/NADH and quinone/quinol) (14, 40, 41). This is different from the role that DMSO serves during anaerobic respiration (anaerobic conditions and dark) when DMSO is the terminal electron acceptor.

The requirement for specific complex I isozymes during photoheterotrophic growth with different carbon sources could reflect changes in the oxidation-reduction state of electron carriers due to the use of different catabolic pathways. To test this hypothesis, we sought to predict or measure the quinol/quinone and NAD^+^/NADH ratios (complex I substrates) found in wild-type cells grown photoheterotrophically with different carbon sources (Fig. 2D). When we used the R. sphaeroides metabolic model (6, 18) to predict quinol/quinone ratios in wild-type cells, we found that photoheterotrophic growth on lactate and succinate is predicted to produce higher (more reduced) quinol/quinone ratios compared to photoheterotrophic growth on fumarate, malate, and pyruvate (Fig. 2D; see also Table S2 in the supplemental material). These relative values reflect both the redox state of the provided carbon substrate (lactate, 0; succinate, +2; pyruvate, +2; malate, +4; fumarate, +4) and the way in which the substrate is metabolized (e.g., enzymes such as succinate and lactate dehydrogenase produce quinol as a product of substrate oxidation). We also directly measured the NAD^+^/NADH ratio when wild-type cells were grown photoheterotrophically with different carbon sources. We found that wild-type cells show a range of NAD^+^/NADH ratios during photoheterotrophic growth on different carbon sources (Fig. 2D), despite having similar growth rates in these media (Fig. 2B). For example, the NAD^+^/NADH ratio of wild-type cells grown on succinate (69:1) is higher than that of cells grown on lactate (21:1) or fumarate (16:1) (Fig. 2D). After considering the impact of carbon catabolism on electron carrier ratios, it predicts that complex I_A_ is important for photoheterotrophic growth when quinol/quinone ratios are higher (more reduced, e.g., succinate and fumarate), while complex I_E_ is important when cells have a lower NAD^+^/NADH ratio (more reduced, e.g., lactate and fumarate). A model predicting the different functions of these isozymes during photoheterotrophic growth on different carbon sources is presented in Discussion (see Fig. 8).

### The complex I_A_ isozyme is important for H_2_ production.

In R. sphaeroides, the nitrogenase enzyme is the only source of H_2_ production, and this system is activated during photoheterotrophic growth when only a poor nitrogen source, such as glutamate, is available (16, 42). We found that *nuoA* transcript abundance from both complex I operons increased under H_2_-producing conditions (photoheterotrophic growth with glutamate as the nitrogen source) (Fig. 3). This observation led to the hypothesis that complex I synthesizes NADH during photoheterotrophic growth, which can serve as a source of electrons for H_2_ production.

To test the function of complex I in H_2_ production, we compared the abilities of wild-type cells and complex I mutant strains to produce this gas. For these experiments, R. sphaeroides strains were grown photoheterotrophically with glutamate as the sole nitrogen source to induce nitrogenase expression and with DMSO to correct for growth defects between wild-type and complex I mutant strains. Control experiments show that the addition of DMSO does not compete with nitrogenase for reductant, as wild-type cultures produce the same amount of H_2_ in the presence or absence of this electron acceptor (16).

**FIG 4:**
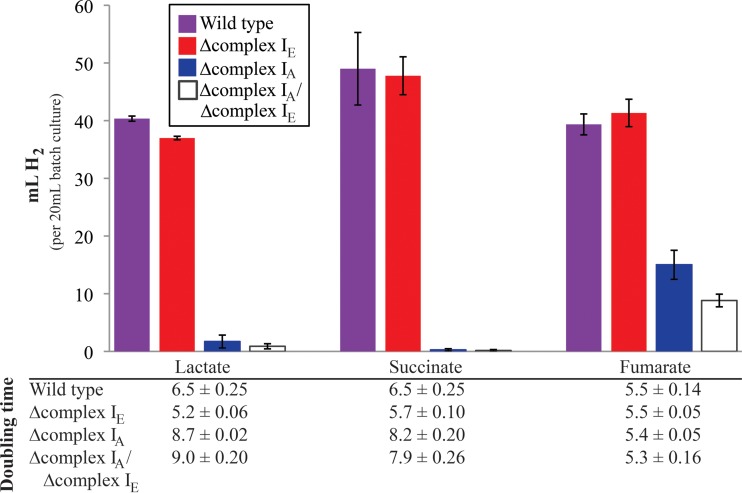
H_2_ production by wild-type and complex I mutant strains grown photoheterotrophically with the indicated carbon sources. All cultures were grown with glutamate as the nitrogen source and the external electron acceptor DMSO (100 mM). Total gas production by each culture was measured and was assumed to be 90% H_2_, as has been previously described (26). The specific H_2_ composition for measurable amounts of gas can be viewed in Table S3 in the supplemental material. The bar graph shows data from ≥4 replicates and includes standard errors. The table below reports the doubling time of each strain (3 replicates, including standard errors) during H_2_-producing conditions (anaerobic in the light, with glutamate as the nitrogen source).

We found that wild-type and Δcomplex I_E_ strains produce the same amount of H_2_ (Fig. 4; see also Table S3 in the supplemental material) when grown photoheterotrophically with succinate, lactate, or fumarate as the sole carbon source. In contrast, the Δcomplex I_A_ and the double mutant strains produced less (fumarate) or essentially no (succinate or lactate) H_2_ under the same conditions (Fig. 4). These data lead us to conclude that complex I_A_ is necessary or important for H_2_ production while the complex I_E_ enzyme does not make a significant contribution to H_2_ production under any of the conditions that we tested. A model explaining the different contributions of each isozyme to nitrogenase-mediated H_2_ production is presented in Discussion (see Fig. 8).

### Additional impacts of loss of complex I_A_ function under anaerobic conditions.

**FIG 5:**
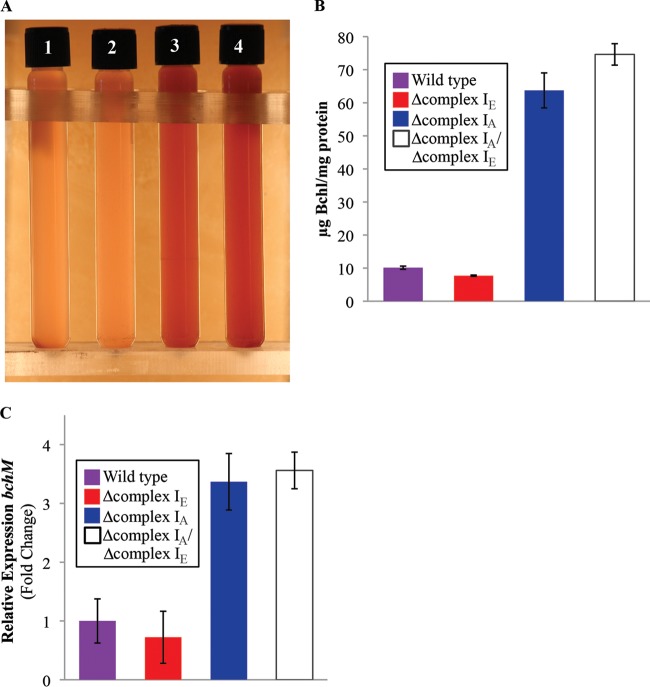
Loss of complex I_A_ increases bacteriochlorophyll levels. (A) Photoheterotrophic cultures (succinate at the carbon source, supplemented with 100 mM DMSO) show different pigmentation in the lanes: 1, wild type; 2, Δcomplex I_A_ strain; 3, Δcomplex I_E_ strain; 4, Δcomplex I_A_/Δcomplex I_E_ strains. (B) Quantification of bacteriochlorophyll (Bchl) in wild type and complex I mutant strains. Data are from 3 replicates, and standard errors are shown. (C) Transcript abundance of the bacteriochlorophyll synthesis gene, *bchM*, in wild-type and complex I mutant strains grown photoheterotrophically (succinate at the carbon source, supplemented with 100 mM DMSO). Data are from 3 replicates, and standard errors are shown.

**FIG 6:**
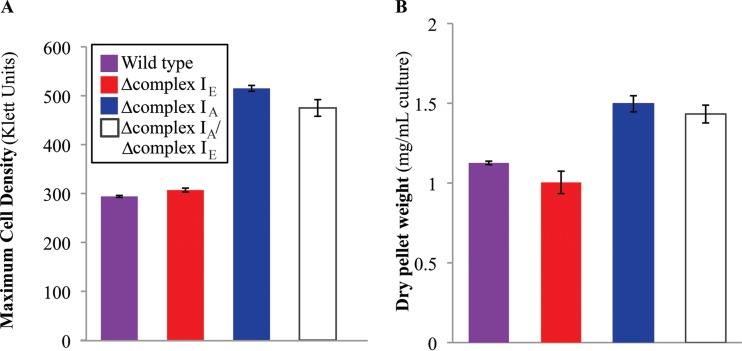
Increased biomass production in the complex I_A_ mutant. (A) Maximum cell density of wild-type and complex I mutant strains grown photoheterotrophically with succinate as the carbon source and supplemented with DMSO. Data are from 5 replicates, and standard errors are shown. (B) Dry weight of wild-type and complex I mutant strains grown photoheterotrophically (succinate as the carbon source, supplemented with DMSO) harvested at maximum cell density (see panel A). Data are from 3 replicates, and standard errors are shown.

In the course of these experiments, we observed other phenotypes when some complex I mutants were grown anaerobically. For example, the Δcomplex I_A_ and double mutant strains had different pigmentation from that of the wild-type and Δcomplex I_E_ strains when grown photoheterotrophically with succinate as the carbon source in the presence of DMSO (Fig. 5A). As expected by the altered pigmentation, we found that the Δcomplex I_A_ and the double mutant strains produced significantly more bacteriochlorophyll than did the wild-type and Δcomplex I_E_ strains under the same conditions (Fig. 5B). In addition, qRT-PCR indicates that this increased pigmentation is associated with elevated transcription of one known pigment (bacteriochlorophyll) biosynthetic gene, *bchM*, in the Δcomplex I_A_ and the double mutant strains (Fig. 5C). We also observed that the Δcomplex I_A_ and the double mutant strains achieve ∼1.5 times the maximum cell density (Fig. 6A) and produce ∼50% more biomass (Fig. 6B) compared to the wild-type or Δcomplex I_E_ strain under the same conditions. Based on what is known about the control of pigment production in R. sphaeroides, we propose that the phenotype of cells lacking complex I_A_ is due to alterations in the oxidation-reduction state of electron carriers (see Discussion).

### Complex I_A_ function is needed for normal aerobic growth.

**FIG 7:**
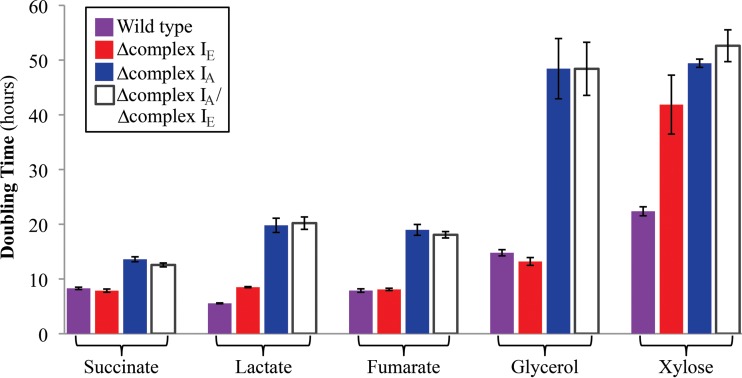
Aerobic growth rates of wild-type and complex I mutant strains. Doubling times of wild-type and complex I mutant strains grown aerobically by shaking in 96-well plates with the indicated carbon sources. Data are from 3 replicates, and standard errors are shown.

In other tested bacteria, loss of complex I activity produces a significant growth phenotype only under anaerobic conditions (13, 14, 23, 38, 43). When we tested the ability of the R. sphaeroides complex I mutant strains to grow aerobically in a 96-well plate format, we found that the doubling time of the Δcomplex I_E_ strain was similar to that of wild-type cells under aerobic conditions with most of the tested carbon sources (Fig. 7). It is not surprising to find that the complex I_E_ isozyme is not required under aerobic conditions since transcripts from this operon are low when cells are grown in the presence of O_2_ (33, 35, 36). However, in the same 96-well aerobic screening conditions, we found that Δcomplex I_A_ and the double mutant strains grew more slowly than wild-type cells on all tested carbon sources (Fig. 7). Control experiments indicate that the defects in the aerobic growth rates of the complex IA and double mutant strains were similar in shake flask cultures (data not shown). Thus, we conclude that the complex I_A_ plays a previously unrealized role under aerobic conditions in R. sphaeroides. This differs from the situation in the closely related bacterium R. capsulatus, where loss of its single complex I_A_-like enzyme is not reported to have significant impact on aerobic growth rates (14, 43).

## DISCUSSION

Complex I is a conserved enzyme that connects the oxidation and reduction of electron carriers (NADH and quinone) to the formation or consumption of the PMF (2). While the role of complex I in the mitochondrial aerobic respiratory chain is well documented (44), its function in the diverse energetic schemes of bacteria is largely unexplored. This work sought to understand the contribution of complex I function to R. sphaeroides. This is a particularly important system to study because the genome of this bacterium encodes two phylogenetically distinct complex I isozymes and it is not predicted to contain other types of NADH dehydrogenases (e.g., NDH-2 [20]). We were also able to take advantage of the wealth of knowledge on R. sphaeroides electron transport chains to interpret the properties of strains lacking one or both complex I isozymes. The major findings and new questions derived from this work are summarized below.

### Complex I is central to R. sphaeroides energy metabolism.

The single complex I of E. coli (a complex I_E_ homologue) is important for anaerobic fumarate and DMSO respiration (13), while the single complex I_A_ homologue in R. capsulatus is required for photoautotrophic and photoheterotrophic growth (14, 43). In contrast, when we analyzed strains containing in-frame *nuoG* deletions from one or both complex I operons, we found that individual complex I isozymes in R. sphaeroides are important for normal aerobic growth and required for all tested anaerobic growth modes (in the absence of DMSO as an electron sink). Indeed, the growth defects of the R. sphaeroides complex I double mutant strain under all tested aerobic and anaerobic conditions expand the role of this enzyme in bacteria.

We also predict that R. sphaeroides complex I has specific roles during different modes of growth. For example, complex I is important for growth by aerobic respiration, since the double mutant strain grows about twice as slowly as the wild type on all tested carbon sources. In the presence of O_2_, the complex I_E_ operon is not expressed (33), but the role of complex I_A_ during aerobic respiration is likely in NADH oxidation. There are likely other enzymes that oxidize NADH (e.g., transhydrogenase) or generate a PMF (e.g., cytochrome *bc*_1_, quinol, or cytochrome *c* oxidase) in the absence of complex I_A_ (6, 45, 46). However, the increased aerobic doubling time of complex I_A_ mutants relative to wild-type cells could reflect the inability of these other enzymes to totally substitute for complex I function.

Our data also show that complex I_A_ was required for dark anaerobic DMSO respiration (conditions under which DMSO is the terminal electron acceptor). We propose that complex I_A_ functions to oxidize NADH under these conditions and likely makes a significant contribution to the PMF. Generation of a PMF would be an important role for complex I_A_ under these conditions since R. sphaeroides DMSO reductase is not a bioenergetic enzyme (17, 47). We also found that complex I_A_ was required for photoautotrophic growth, in agreement with findings from R. capsulatus, in which complex I is proposed to be required for synthesizing reductant (NADH) for CO_2_ fixation (4, 14, 15). However, this function apparently cannot be provided by complex I_E_, which remains intact in the complex I_A_ mutant strain. Lastly, both complex I isozymes were found to contribute to photoheterotrophic growth, depending on the provided carbon substrate.

### Complex I is required to maintain a cellular redox state during photoheterotrophic growth.

We found that the photoheterotrophic growth defects of the single and double complex I mutants strains were partially or fully rescued by addition of the external electron acceptor, DMSO (conditions under which DMSO serves as an “electron sink” to recycle excess reductant [Fig. 2C]). When R. sphaeroides grows photoheterotrophically, electrons derived from the photosynthetic electron transport chain are shuttled between the light-excited reaction center and the cytochrome *bc*_1_ complex (via quinone and cytochrome *c*_2_), which generates a PMF (Fig. 2A) (48). During photoheterotrophic growth, electrons may also enter the quinone pool by catabolism of an organic carbon source (e.g., succinate dehydrogenase). The reduction of the quinone pool via both light energy capture and carbon catabolism during photoheterotrophic growth likely increases the need to oxidize quinol in order to create the quinone that is required for photosynthetic electron transfer. In the photosynthetic bacterium R. capsulatus, NADH synthesis by its single complex I enzyme has been proposed to prevent overreduction of the quinone pool (Fig. 2A) (14, 15). DMSO reductase can accept electrons from quinol (39), so the ability of this external electron acceptor to repair the photoheterotrophic growth defects of complex I mutants supports the hypothesis that complex I functions to prevent overreduction of the quinone pool (or other electron carrier pools) under these conditions (Fig. 2A). Bioinformatic analysis of the bacterial genome database predicts that all sequenced genomes of purple photosynthetic bacteria encode at least one complex I_A_ homologue (4). Thus, the function of complex I_A_ to prevent overreduction of the quinone pool during photoheterotrophic growth may be conserved across purple photosynthetic bacteria. In contrast, intact complex I operons are not often found in the genomes of other phototrophic bacteria (4), so these species (which often produce reduced ferredoxin as a product of light energy capture) may not need this enzyme to prevent overreduction of the quinone pool.

### Different roles for individual complex I isozymes.

Analysis of R. sphaeroides mutants containing one intact complex I operon also allowed us to dissect the physiological function of the phylogenetically distinct isozymes. We found that the canonical alphaproteobacterial complex I enzyme, complex I_A_, has more functions in R. sphaeroides than the complex I_E_ enzyme. We found that complex I_A_ is important for aerobic respiration and required for anaerobic DMSO respiration, photoautotrophic growth, or photoheterotrophic growth on specific carbon sources. In contrast, the complex I_E_ isozyme, which was likely acquired from a gammaproteobacterium (4), is only required for photoheterotrophic growth on select carbon sources. We expected to find that each complex I isozyme contributes to photoheterotrophic growth because transcripts from both *nuo* operons are present under these conditions (33, 34). However, a role for individual complex I isozymes during photoheterotrophic growth on different carbon sources was unexpected based on the lack of carbon source-dependent alterations in *nuoA* transcript levels (Fig. 3) or predictions of the R. sphaeroides metabolic model (6, 18). Further analysis of the complex I mutants should improve our ability to model R. sphaeroides energetic and metabolic pathways.

**FIG 8:**
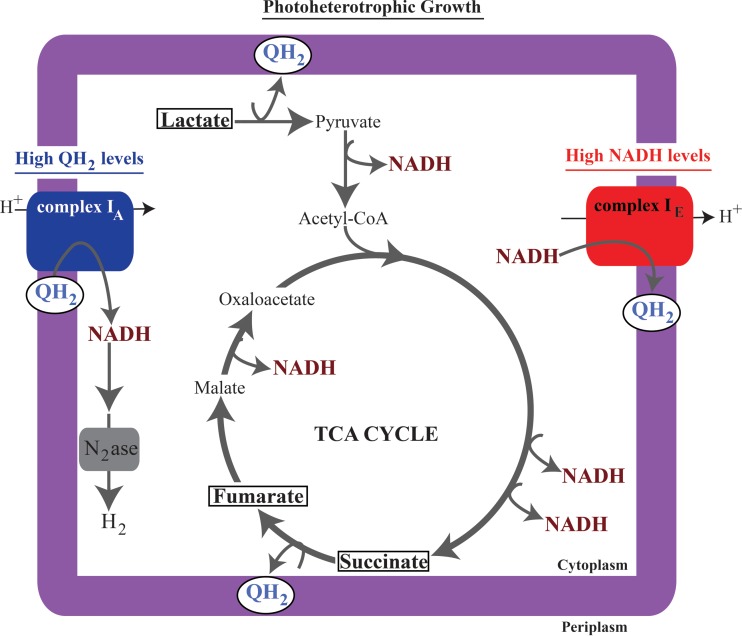
The proposed contribution of complex I isozymes during photoheterotrophic growth. We predict that lactate catabolism produces high levels of both quinol (49) and NADH (Fig. 4), succinate catabolism produces high levels of quinol (6, 18), and fumarate catabolism produces high levels of NADH (Fig. 4). To explain the properties of complex I mutants, we propose that complex I_E_ is important for growth on carbon sources that produce high levels of NADH, where it oxidizes NADH to maintain redox state. Alternatively, we propose that complex I_A_ is important for growth on carbon sources that produce quinol, where it functions to synthesize NADH, thereby preventing overreduction of the quinone pool and producing the cellular reducing power that is shuttled into nitrogenase (N_2_ase)-mediated H_2_ production.

If one considers the products of carbon catabolism, it is possible to propose a model to explain why individual complex I isozymes might be important for photoheterotrophic growth when metabolizing specific carbon sources (Fig. 8). Complex I_A_ is required for photoheterotrophic growth on carbon sources (succinate and lactate), where catabolism is predicted to produce a more reduced (higher) quinol/quinone ratio (Fig. 2B and D). Under these conditions, the first step in carbon catabolism is predicted to directly produce quinol via succinate or lactate dehydrogenase (49) (Fig. 8). Unlike aerobic or anaerobic respiratory growth conditions, there is no terminal electron acceptor during photoheterotrophic growth; thus, we predict that complex I_A_ functions to synthesize NADH during photoheterotrophic conditions in order to prevent overreduction of the quinone pool (Fig. 8). In further support of this model, the Δcomplex I_A_ mutant strain is able to grow photoheterotrophically with pyruvate as a carbon source but not with lactate. One possible relevant difference in the catabolism of these substrates is that pyruvate metabolism produces less quinol (growth on pyruvate bypasses the quinol-producing lactate dehydrogenase [Fig. 8]), and with a more oxidized (lower) quinol/quinone ratio complex I_A_ is not required for growth. Conversely, we find that complex I_E_ is required or important for photoheterotrophic growth when there is a more reduced (lower) NAD^+^/NADH ratio, namely, in cells using lactate or fumarate as a carbon source (Fig. 2B and D). Thus, we predict that complex I_E_ functions to oxidize NADH during photoheterotrophic growth on carbon sources that produce relatively low NAD^+^/NADH ratios relative to other substrates. Complex I_E_ would function to regenerate the NAD^+^ required for other catabolic processes. Therefore, we propose that both isozymes contribute to photoheterotrophic growth, where they help maintain redox state by modulating either the quinol/quinone or NAD^+^/NADH pools (Fig. 8).

### Possible functional differences between the complex I_A_ and complex I_E_ isozymes.

While both complex I isozymes are presumably capable of performing NADH oxidation and synthesis, there may be differences between the complex I isozymes that explain their predicted relative roles in R. sphaeroides photoheterotrophic growth. For instance, biochemical analysis of complex I_A_-like enzymes from other bacteria suggest that these enzymes contain two pyridine nucleotide binding sites: one for NADH and one for NAD^+^ (4, 50–54). In contrast, only a single NADH binding site has been found in the structures of bacterial complex I enzymes (12, 55). To date, all the published structural data on bacterial complex I have been derived using enzymes that are closely related either to R. sphaeroides complex I_E_ (E. coli) or to the enzyme from Thermus thermophilus (which contains a clade D complex I, described in reference 4). Thus, the presence of different nucleotide binding sites in complex I_A_ and complex I_E_ isozymes may impact the relative ability of these two enzymes to synthesize or oxidize NADH *in vivo*. It is also worth noting that all lithotrophs, many of which are predicted to use complex I for NADH synthesis (56–58), have a complex I_A_-like or clade B enzyme (4). Purple photosynthetic bacteria, such as R. sphaeroides, and lithotrophs require complex I for NADH synthesis, so clade A and B complex I enzymes may have evolved to perform NADH synthesis more efficiently than other complex I enzymes (e.g., clade E complex I in E. coli is predicted to perform NADH oxidation during anaerobic respiration [13]). Additional kinetic and structural studies of each R. sphaeroides complex I isozyme, using proteins prepared from mutants described in this paper, could be used to test if there are significant kinetic or substrate binding properties of the complex I_A_ and complex I_E_ isozymes.

In both mitochondria and the alphaproteobacterium Paracoccus denitrificans, complex I has been reported to form supercomplexes with other bioenergetic enzymes (8, 59–62). Similar experiments in the gammaproteobacterium have failed to find evidence for supercomplexes of its complex I (a complex I_E_ homologue) with quinol oxidase or other membrane electron transport complexes (63, 64). Mitochondria, P. denitrificans, and R. sphaeroides each use cytochrome *bc*_1_ complexes in their electron transport chains, while E. coli does not (5, 7, 8, 48, 59–62). Thus, additional experiments are needed to test if the ability of complex I_A_ or complex I_E_ to form supercomplexes with the cytochrome *bc*_1_ complex or other electron transport complexes can partly explain the proposed differences in the relative efficiency of NADH synthesis and oxidation by different complex I isozymes that we predict *in vivo*.

### Complex I provides reductant for other pathways.

We also found that complex I_A_ was required for wild-type levels of nitrogenase-mediated H_2_ production (16, 65) while complex I_E_ is dispensable for this process. These findings provide additional support for the relative importance of individual isozymes in NADH synthesis (complex I_A_) and oxidation (complex I_E_) under photoheterotrophic conditions (Fig. 8). It is not known how the Δcomplex I_A_ and double mutant strains were able to produce H_2_ when growing photoheterotrophically with fumarate as the carbon source. In this regard, it might be worth noting that the use of fumarate as a carbon source is among conditions under which the NAD^+^/NADH ratio is most reduced (lowest value [Fig. 2D]), so perhaps some NADH produced via fumarate catabolism is used to support the small amount of H_2_ produced in the presence of this carbon source. While NADH is unable to directly reduce nitrogenase, the Rnf complex is thought to use the PMF to drive electron transfer from NADH to ferredoxin, which is capable of reducing nitrogenase (66).

We also found that the loss of complex I_A_ led to several other unexpected phenotypes. For example, the complex I_A_ mutant grew more slowly photoheterotrophically but had increased levels of pigment (bacteriochlorophyll) and produced more biomass than wild-type cells grown under the same conditions. Our data predict that complex I_A_ maintains the redox state by synthesizing NADH during photoheterotrophic growth, so the loss of this enzyme would likely alter the redox state of both the quinone and pyridine nucleotide pools under these conditions in ways that may help explain these phenotypes. First, changes in the redox state of the quinone pool are thought to alter transcription of many energy-producing and -consuming pathways (including genes involved in bacteriochlorophyll biosynthesis, e.g., *bchM*) via the two-component regulatory system RegB/RegA (PrrB/PrrA), potentially resulting in increased pigment production in the complex I_A_ mutant (67, 68). Second, because we predict that complex I_A_ is a source of NADH for pathways such as CO_2_ fixation and nitrogenase-mediated H_2_ production, the loss of this enzyme may allow cells to route more reducing power into biomass pathways. Additional experiments are required to test if gene expression and carbon/electron partitioning are altered in complex I_A_ mutants in order to better understand the cause for these phenotypes.

### Conclusions.

This study showed that bacterial complex I can serve multiple, previously unrecognized functions. We found that R. sphaeroides complex I activity is important for aerobic respiration and required for anaerobic DMSO respiration, photoautotrophic growth, and photoheterotrophic growth (in the absence of an external electron acceptor). We predict that the alphaproteobacterial complex I_A_ in R. sphaeroides functions to oxidize NADH during aerobic and anaerobic respiration and to synthesize NADH during phototrophic conditions. Our data also provide insight into the relative function of the phylogenetically distinct R. sphaeroides complex I enzymes (complex I_A_ and complex I_E_) in maintaining the cellular redox state during photoheterotrophic growth. We propose that the relative importance of either isozyme under these conditions is linked to a function of complex I_A_ for NADH synthesis and complex I_E_ for NADH oxidation. The canonical alphaproteobacterial complex I isozyme (complex I_A_) was also shown to be important for routing electrons to nitrogenase-mediated H_2_ production, while the horizontally acquired enzyme (complex I_E_) was dispensable in this process. These findings demonstrate that the multiple complex I isozymes found in a given bacterium are not necessarily redundant, suggest that the single complex I enzyme in most species has evolved to suit the energetic needs of its host, and highlight the need for additional studies to explore the functions of the different classes of complex I enzymes across the bacterial phylogeny (4).

## Supplementary Material

Supplemental material
