# Supplementary material for: Different Functions of Phylogenetically Distinct Bacterial Complex I Isozymes
Source: J Bacteriol. 2016 Mar 31;198(8):1268–80. doi: 10.1128/JB.01025-15 (PMC4859585; doi:10.1128/JB.01025-15)
Supplement: Supplemental material [file supp_198_8_1268__index.html]

Supplemental material 

# Different Functions of Phylogenetically Distinct Bacterial Complex I Isozymes

## Supplemental material

- Supplemental file 1 -

  Table S1, complex IA and complex IE*nuoA* expression in mutant strains

  Table S2, predicted metabolic flux for quinol-producing reactions in wild-type cells

  Table S3, gas composition of strains

  PDF, 102K
