# Supplementary material for: Different Functions of Phylogenetically Distinct Bacterial Complex I Isozymes
Source: J Bacteriol. 2016 Mar 31;198(8):1268–80. doi: 10.1128/JB.01025-15 (PMC4859585; doi:10.1128/JB.01025-15)
Supplement: Supplemental material [file JB.01025-15_zjb999094003so1.pdf]

Table S1. Complex I_A and complex I_E *nuoA* expression in photoheterotrophically-grown mutant strains

**Strain**	**Carbon source**	**DMSO** (100mM)	**Complex I_A *nuoA*[a,b] (Relative expression)**	**Complex I_E *nuoA*[a.b] (Relative expression)**
Wild type	succinate	-	1.00 ± 0.5	1.00 ± 0.2
Δcomplex I_E	succinate	-	0.97 ± 0.2	0.47 ± 0.5
Δcomplex I_E	succinate	+	0.61 ± 0.3	1.11 ± 0.5
Δcomplex I_A	succinate	+	0.70 ± 0.6	1.06 ± 0.1
Δcomplex I_A/Δcomplex I_E	succinate	+	0.72 ± 0.2	1.00 ± 0.1
Δcomplex I_E	fumarate	-	0.45 ± 0.4	0.38 ± 0.3
Δcomplex I_A	fumarate	-	0.16 ± 0.2	0.29 ± 1.3
Δcomplex I_E	fumarate	+	0.36 ± 0.2	0.35 ± 0.4
Δcomplex I_A	fumarate	+	0.34 ± 0.5	0.24 ± 0.4
Δcomplex I_A/Δcomplex I_E	fumarate	+	0.30 ± 0.2	0.25 ± 0.4

a: values were averaged from 3 replicates, and show standard error
b: transcript levels were normalized using housekeeping gene *rpoZ*

Table S2: Predicted metabolic flux for quinol-producing reactions in wild type cells grown photoheterotrophically on different carbon sources

Carbon source	Lower bound quinol-producing flux[a]	Upper bound quinol-producing flux[a]
Lactate	-0.004	5.647
Succinate	1.721	3.647
Fumarate	-0.004	1.647
Malate	-0.004	1.647
Pyruvate	-0.004	2.981

a: the model provides a predicted range (lower and upper bound) for the metabolic flux of quinol production. A quinol flux that is > 0 represents a condition where more quinol is produced than quinone, and a value < 0 represents a condition where more quinone is produced than quinol, during catabolism of the carbon source. A more negative value indicates a greater capacity for quinone production, while a more positive value indicates a greater capacity for quinol production.

Table S3. Gas composition of strains grown photoheterotrophically under nitrogen-fixing conditions in the presence of DMSO

Strain	Carbon source	Gas composition (% H2)[a]
Wild type	succinate	93.1 ± 3
Δcomplex I_E	succinate	96.6 ± 4
Wild type	fumarate	92.2 ± 4
Δcomplex I_E	fumarate	90.5 ± 3
Δcomplex I_A	fumarate	83.6 ± 8
Δcomplex I_A/Δcomplex I_E	fumarate	84.8 ± 11

a: H_2 composition was measured from 3 replicates, and the value includes standard error.
